# A study on the quality of study skills of newly-admitted students of Fasa University of Medical Sciences

**Published:** 2014-01

**Authors:** FATEMEH SHAHIDI, HAMID REZA DOWLATKHAH, ABOLGHASEM AVAND, SEYED REZA MUSAVI, ELAHEH MOHAMMADI

**Affiliations:** 1 Education Development Center, Fasa University of Medical Sciences, Fasa, Iran;; 2 English Language Department, Fasa University of Medical Sciences, Fasa, Iran;; 3 Fasa University of Medical Sciences, Fasa, Iran; 4 Shiraz University of Medical Sciences, Shiraz, Iran

**Keywords:** Skill, Students, Learning

## Abstract

**Introduction**: Some students attribute their academic failure to such factors as low aptitude, unavailability of resources, and bad luck. However, we can dare to say that the most important factor playing a role in academic success is students' little acquaintance with learning and study skills. This study aimed at examining the quality of study skills in newly-admitted students of Fasa Medical University so that the results can be used in holding teaching courses in study skills.

**Methods**: The present study is a cross-sectional descriptive study. The sampling was done of all the newly-admitted students in the first semester of 2012 academic year including 94 students of Medicine, Nursing and Laboratory Technology. The data were collected through a questionnaire, consisting of two parts. The first part included items on demographic information of the subjects (such as sex, field of study, number of hours dedicated to studying, student's rank in Konkour, and the National University entrance exam. The second part was composed of 19 special items on such domains as 'Time management', 'Concentration', 'Class note-taking', 'Studying' and 'Taking exams' with 4, 5, 4, 3, 3 items, respectively. The checklists were filled in using Likert Scale. The collected data was then analyzed using an SPSS 14, through which descriptive statistics as mean, standard deviation and multiple regressions were obtained. Moreover, the data were analyzed using Independent Sample t and ANOVA tests.

**Results**: The results showed that the range of the students' study skills was 2.35, being rather below the normal level; the highest mean belonged to 'concentration' (2.56), but the lowest mean was that of 'time management' (2.05).Through ANOVA test, it was also shown that there was no significant difference between the students of Medicine, Nursing and Laboratory Sciences regarding their scores on ‘study skills’ as (p=0.646). In addition, through independent sample t-test, it was shown that there was no significant difference between the subjects’ ‘sex’ and ‘study skills’ as the p-value was 0.584. On the other hand, through multiple regressions, the results indicated that there was a significant difference between 'taking exams’ and ‘studying’ (p=0.003), between ‘class note-taking’ and ‘'taking exams’ (p=0.004), between ‘concentration' and ‘'taking exams’ (p=0.002), and between ‘time management’ and 'taking exams’ (p=0.001).

**Conclusion**: Regarding the very important role of study skills in learning, it is recommended that 'study skills' and 'study habits' courses be included in the students' curriculum formally or implemented as workshops for students.

## Introduction

The students' unfamiliarity with study skills is a problem of Iranian educational systems. It seems that students' lack of interest and enthusiasm in reading while studying lessons on the one hand and their inability to recall the lessons studied on the other hand result from their poor study skills and reading strategies. Reading is pleasing and effective providing that students do know how to study certain textbooks. Statistics and experiments show that a high percentage of high school students and freshmen, not having learned to study scientifically, study their lessons aloud, just retain, and memorize the materials; however, they are not thinking of recalling and reviewing their lessons (1).

Since it is a difficult task to learn and retain a considerable volume of up-to-date and specialized information the students confront in an academic year which takes them a lot of time and requires them to have a planning, it is obvious that lack of appropriate study skills and habits not only leads to the students' waste of time, energy and their tendency toward bad study habits, but also results in their backwardness which in turn causes confusion and anxiety. They in effect influence the students' performance in exams (2).

The quality and quantity of learning are influenced by different factors. Although such various variables as general intelligent quotient, physical and mental health, motivation, interest in a subject, relaxation, teaching and teaching-aid facilities and students' cognitive capabilities all affect the level of learning and recalling the contents of a lesson, psychologists have observed that the most contributing factor in students' performance, at least while studying at a university, is general study skills in learning and recalling the lessons studied (3).

Studying lessons is a major factor in development of research and educational systems. Therefore, making attempts in improving study conditions and improving reading strategies are considered to be one of the significant educational measures (4).

Studying, as a mental processing, has its own principles and specific conditions. Studying conditions are such issues through which one can expect to have a more useful and efficient studying by knowing and applying them? A fruitful studying depends on the two following factors: 1) the reader's interest in the material or the textbook being studied and 2) his or her skillful use of reading strategies or study skills. The first factor motivates the reader to study more which in turn leads to betterment of the use of study skills (5). Accordingly, the use of better skills facilitates studying and makes it faster and funnier. Therefore, the reader is more interested in his study; making him study more and not preventing him or her from studying the material that is a must to study (6).

In Iranian educational system, students are differently evaluated during and at the end of a semester through various tests and exams; however, they are not given any hints about how to study and take exams. The students spend a lot of time studying; still, they do think little about the improvement of their study skills. In fact, they follow what they are used to, following the skills insufficiently learned in high schools. Having been admitted at universities, students spend lots of time adjusting themselves to the new environment or actually student life. It takes them several semesters or even years, namely at the end of their graduation from university, when they have learned a few desirable study skills. Most of the students even at the end of their study have not yet learned study skills sufficiently. Learning the contents of the textbooks and scientific information does not stop by merely studying, but it should lead to comprehension. In other words, the contents of the textbooks require the students' active reading in order to get them prepared for their comprehension (7).

The studies carried out on cognitive domain indicate that study skills and learning are improved as learning and students' academic performance are facilitated (8). Research has indicated that the students' participation in seminars on study skills increases the rate of their retention (9-12).

It is doomed to be essential for the newly-admitted students in such Universities as York in Canada, Ferrum College, Berkley in California, Cook Consultation Center in Virginia, Dartmouth and some other teaching institutes to acquire knowledge on study skills and reading strategies (13-16).

Among Iranian researchers, Shams has suggested that students' problems in learning skills and unfamiliarity with efficient reading strategies contribute to their academic failure (17). In another study by Aminian carried out in Yazd Medical University, it was found that successful students had made use of more effective study skills in their lessons (18).

One of the reasons why students achieve little is their ignorance of study skills and related strategies. Academic development is one of the major goals of educational institutes as students' academic performance determines the academic development at every level. In fact, academic performance is influenced by various variables among which study habits and reading strategies can be mentioned (2).

The present study aims at examining the study skills of newly-admitted students at Fasa University of Medical Sciences in 2012 academic years.

## Methods

This is a cross-sectional descriptive study. The subjects of this study consist of all the newly-admitted students in the first semester of 2012 academic year; 94 students of Medicine, Nursing and Laboratory Technology participated in the study which was carried out during the first week of the 1st semester. A researcher-made questionnaire was used to collect the data. The validity of the questionnaire was confirmed by the experts. Also, its reliability was calculated using Cronbach’ s alpha Formula (α=0.689). The questionnaire consisted of two parts. The first part included items on demographic information of the subjects (such as sex, field of study, number of hours dedicated to studying, and the student's rank in Konkour, Iranian University Entrance Exam. The second part was composed of 19 special items on such domains as 'Time Management', 'Concentration', 'Class note-taking', 'studying' and 'Taking exams' with 4, 5, 4, 3, 3 items, respectively. The mean of the responses to each item was considered as the score of that skill. The mean for study skill was derived from the mean of the responses to 19 items of the questionnaire. The items were scored through five-point Likert scale as 'Totally disagree' and 'Totally agree' with one to five scores. Level normal scores are 2.5 out of 5. The mean scores indicate the subjects' study habits and skills. 

The collected data were then analyzed using an SPSS 14 (SPSS Inc, Chicago, IL, USA) through which such descriptive statistics as mean and standard deviation were obtained. Moreover, the data were analyzed by Independent Sample t, ANOVA, and Multiple Regression Tests. 

## Results

All 94 questionnaires distributed among the subjects were returned to the researchers. The obtained data show that 41 subjects were male (43.5%) and 53 subjects were female (56.5%). The mean and standard deviation of scores on students' study skills for each field of study are shown in Table 1.

**Table 1:** The mean and the standard deviation of scores for each field of study

**Students' major**	**No.**	**Mean±SD**	**p**
**Medicine**	40	2.40±0.55	0.646
**Nursing**	33	2.31±0.56	
**Lab technology**	21	2.39±0.44	

With regard to the use of study skills among students in different fields of study, the results showed that the students of medicine achieved the highest score (mean=2.40), the students of laboratory technology ranked the second (mean=2.39), and the students of nursing ranked the third (mean=2.31).

The calculated p-value (0.646) through ANOVA Test which is significantly more than 0.05 showed that there was no significant difference in using study skills among students of Medicine, Nursing and Laboratory Technology.

Table 2 shows the subjects' scores on such variables as 'Time management', 'Concentration', 'Class note-taking', 'Studying', 'Taking exams', and 'Study skills'.

**Table 2:** The mean and the standard deviation of scores on students' study skills

**Study skills**	**Mean±SD **
**Time management**	2.05±0.702
**Concentration**	2.56±0.618
**Class note-taking**	2.43±0.504
**Studying**	2.43±0.923
**Taking exams**	2.28±0.775
**Study skill**	2.35±0.525

The results obtained by statistical analysis and calculation of the means in different domains with respect to the subjects' use of study skills show that the highest mean belongs to 'Concentration'( the mean is 2.56) and the lowest one goes to 'Time management'(the mean is 2.05). Furthermore, the overall mean indicates that the subjects' use of study skills is 2.35 which are rather below the average level. The following table shows the mean scores of the subjects' study skills in terms of their sex.

**Table 3:** The mean, the standard deviation, p-value of scores on students' study skills according to the subjects' sex

**Study skills**	**Sex**	**N**	**Mean±SD**	**p **
**Time management**	Male	40	2.15±0.69	0.21
	Female	52	1.96±0.71	
**Concentration**	Male	40	2.52±0.68	0.46
	Female	52	2.61±0.57	
**Class note-taking**	Male	40	2.42±0.50	0.79
	Female	52	2.45±0.51	
**Studying**	Male	40	2.36±0.87	0.51
	Female	52	2.49±0.97	
**Taking exams**	Male	40	2.23±0.84	0.49
	Female	52	2.34±0.73	
**Study skill**	Male	40	2.33±0.51	0.74
	Female	52	2.37±0.54	

The results obtained by statistical analysis and calculation of the means, SD and p-value in different domains of study skills with respect to the subjects' sex indicate that the mean of study skills among male students is 2.33 and that for females is 2.37. The findings of independent sample t-test showed that there was no significant difference between the female and male students in such domains as 'Concentration', 'Class note-taking', 'Studying' and 'Taking exam', respectively. The statistics show that there is no significant difference between the students' sex and the use of study skills )p=0.584(. The relationship between such variables as 'Studying', 'Class note-taking', 'Time management' and 'Concentration' and 'Taking exam' is displayed in Table 4.

**Table 4:** The relationship between such variables as 'Studying', 'Class note-taking', 'Time management' and ‘Concentration’ with ' Taking exams' as the dependant variables

**Skills**	**Mean±SD**	**B cofficient**	**Std. Error**	**Beta cofficient**	**t**	**Sig**
**Time management**	2.05±0.70	0.505	0.090	0.457	5.582	<0.001
**Concentration**	2.56±0.61	0.432	0.109	0.345	3.983	<0.001
**Class note-taking**	2.43±0.50	2.428	0.215	1.580	11.271	<0.001
**Studying**	2.43±0.92	0.427	0.078	0.508	5.470	<0.001

 In order to determine the relationship between such variables as 'Class note-taking', 'Time management', and 'Concentration' and 'Taking exam', the researchers made use of a multivariable regression. The correlation coefficient and determination coefficient were 0.82 and 0.67, respectively. There was a significant difference between such variables as 'Studying', 'Class note-taking', and 'Time management', and 'Taking exam' as the p-value is zero which is significant at 0.05 level. All variables were proved to have effects on taking exams. While ‘class note-taking’ variable was proved to have the greatest effect on students’ taking exams (Beta=1.58), ‘Concentration’ was proved to have the lowest effect on taking exams (Beta=0.345). 

## Discussion

This study was conducted to examine the students' use of study skills. The students were newcomers to Fasa University of Medical Sciences majoring in Medicine, Nursing and Laboratory Technology. In examining the students' scores on study skills in terms of their fields of study, it was revealed that the students of Medicine had the highest score in study skills. However, there was no significant difference between the students of Medicine, Nursing and Laboratory Technology with regard to study skills. Therefore, apart from having skills in studying, the students' success in Iranian University Entrance Exam (Konkour) is affected by other variables which need to be further studied.

Time is the best treasure that a student owns. Based on this study, a major problem of students was lack of time management. In another study by Nourian carried out in Zanjan University of Medical Sciences, the major problems of students were found to be 'Time management', 'Concentration', 'Studying speed', and 'Class note-taking'. 'Time management' was considered to be the students' most important problem. In order to prevent and solve such a problem, it is recommended that the students should be taught how to reduce the pauses between their studying sessions and remove the distractors (19).

In this study, the highest mean score belonged to such skills as 'Concentration', 'Class note-taking', and 'Studying'. As we know, learning rests on concentration. In a study by Hosseini, it was known that there was a significant and positive correlation between concentration rate and academic advancement (12).

Based on the mean scores (2.35) on different domains, it can be concluded that the freshmen students' condition with regard to 'Study Skills' is rather below the normal level. The results of this study as well as those conducted in other Iranian universities indicate that there is a gap between the students' current condition and the ideal one. In studies performed by Kushan & Haidari (2006), Mohammadi (2006), and Feraiduni (2007), the condition of students' study skills was at an average level (11, 20, 21). In addition, their studying approach is not of a high quality. In a study by Hossaini et al. (12), it was claimed that only 30.7% of students were in a good study skill condition. In another study by Sayfuri, the students' study skills condition was assessed to be rather below the normal level (2). Regarding the importance of studying in the process of learning, the students' present condition cannot be considered ideal at all. It seems that the students are not sufficiently provided with essential facilities, learning and teaching conditions so that they can develop and promote their own study skills and study habits. It is recommended that students on admission to any university be intensively taught how to make use of study skills.

In this study, the male students obtained lower scores on such domains as 'Concentration', 'Class note-taking', 'Taking exams', and 'Studying' than female ones.

In another study by Nourian, the male students had lower scores on 'Comprehension', Concentration while Studying', and 'Time management' than female ones (19). However, in this study, the students' scores obtained on 'Time management' were higher than those of female ones. These results are in the same line with those of the studies done by Fehri (22) and However, in a study by Trueman, the female students were in a better condition than male ones on 'Time management' (15).

In this study, it was revealed that there was no significant difference between sex and study skills. The same results had already been obtained by Hossaini et al. (12). In another study by Badeleh et al. (23), however, the use of study skills was significantly higher among female students than male ones.

The present study showed that there was a significant difference between such skills as 'Time management', 'Concentration', and 'Class note-taking' skills. It was known that 'Class note-taking' was the most contributing factor in taking a good exam. In a study by Nneji, most of the students made use of 'Class note-taking' and 'Hand-outs' as important sources for studying their lessons. It was sufficient for them to only listen to the lectures presented by the university teachers (14).

It this study, it was known that 45.1% of the students had intensively studied their lessons just the night before exams. In a study by Rouhani and his colleagues, it was indicated that 65.6% of the students studied at the night before their exams which resulted in increasing anxiety and exhaustion (24).

The present study showed that only 12% of the students had a plan to study while 77.6% of them studied once in a while without any plan in advance. In the study by Ravari et al., it was also ascertained that students do not have any comprehensive plan to budget their time for studying. The students, however, succeed to pass their courses with a fairly good grades; this may be due to the fact that the only criterion for evaluation of students is their grades on the exam. They can get the desired grade without trying very hard or spending a lot of time since there is no ground for them to endeavor for time management (25).

Mohammadi and Dadkhah in their study stated that only 16% of the students had a well-organized plan to study for their final exams while Hossaini et al. found out that 82% of the students had a plan to do so (20). The results of the studies by Steinert showed that by holding Time Management Workshop, the participants’ time management skills, setting goals, and prioritizing their duties were promoted (26). Furthermore, the findings of the study by Haghani and Khadivzadeh show that teaching the students through workshops was effective for promotion of the students’ study skills and learning (27).

## Conclusion

Researchers believe that study skills such as class note-taking, promotion of concentration, time management, accelerating studying ability and preparation for taking exams can be improved. They have come to the conclusion that improving study skills may give rise to academic advancement; therefore, the learners’ motivation enhances. It is recommended that teaching skills and study habits be presented either as a credit or a workshop in the curriculum of the newly-admitted students so that on their arrival they would get acquainted with these skills which certainly leads to better learning. The provision of studying backgrounds as well as encouraging students to look scientifically into studying approaches could be more effective.
